# Traditional Chinese Medicine, the Zishen Yutai Pill, Ameliorates Precocious Endometrial Maturation Induced by Controlled Ovarian Hyperstimulation and Improves Uterine Receptivity via Upregulation of HOXA10

**DOI:** 10.1155/2015/317586

**Published:** 2015-02-22

**Authors:** Qi Gao, Lu Han, Xiumei Li, Xia Cai

**Affiliations:** ^1^Department of Gynecology, Traditional Chinese Medical Hospital of Xinjiang Uygur Autonomous Region, Urumqi, Xinjiang 830000, China; ^2^Department of Morphology Center, Xinjiang Medical University, Urumqi, Xinjiang 830000, China; ^3^Reproductive Center, The First Teaching Hospital of Xinjiang Medical University, Urumqi, Xinjiang 830000, China

## Abstract

Controlled ovarian hyperstimulation (COH) is widely used in assisted reproductive technology (ART), but it often leads to precocious maturation of the endometrium such that it impairs embryonic implantation and limits pregnancy rates. Previous studies have shown the traditional Chinese medicine, the Zishen Yutai pill (ZYP), to be effective in treatment of threatened as well as recurrent miscarriages, and it can improve embryonic implantation rates in patients undergoing IVF treatment. In the present study, the ZYP has been found to ameliorate precocious endometrial maturation in a mouse model of different COH. Molecular evaluations, real-time PCR, relative RT-PCR, Western blotting, and immunohistochemistry have indicated that the ZYP increased the expression of HOXA10, an important marker of uterine receptivity. Elevation of HOXA10 led to further upregulation of its target gene, integrin *β*3, and downregulation of EMX2, two additional markers of uterine receptivity. In this way, the ZYP may mitigate COH-induced precocious maturation of the endometrium and improve uterine receptivity by upregulating HOXA10.

## 1. Introduction

It is estimated that over 4% of all infants born every year in the more developed countries are conceived using assisted reproductive technology (ART) [1]. However, despite years of attempts to improve the efficiency of ART, pregnancy rates still remain moderate and have not increased significantly [1, 2]. It has been suggested that this relatively low pregnancy rate is in large part due to the widespread use of a technique termed “controlled ovarian hyperstimulation” (COH) [3–5]. COH has been developed for the collection of a large number of follicles in order to retrieve mature oocytes. COH is achieved by daily injection of follicle-stimulating hormone (FSH) [6]. To prevent a surge in premature luteinizing hormone (LH) and premature ovulation, gonadotropin-releasing hormone (GnRH) agonist or antagonist is also injected [6]. However, ovarian stimulation often disturbs luteal cycles and induces abnormalities in endometrial development. These abnormalities manifest mainly as precocious maturation of the endometrium, which often leads to frequent glandular and stromal dyssynchrony in the mid-luteal phase [3–5]. Studies have suggested that this advanced maturation of the endometrium may account in large part for the failure of embryonic implantation [3–5]. Embryonic implantation is an essential step in the development of a pregnancy and its failure continues to impede treatment outcomes of ARTs [3]. A key factor determining the success of embryo implantation is the receptivity of the endometrium, which is often impaired by precocious endometrial maturation in ART cycles [3]. This has led to widespread adoption of clinical interventions aimed at improving endometrial receptivity in ART cycles [3].

The Zishen Yutai pill (ZYP) is a TCM formula that contains* Cuscuta sinensis* Lam., Fructus Amomi, Radix Rehmanniae Preparata, ginseng, Herba Taxilli, Colla Coriiasini, Polygonum Multiflorum, Artemisiae Argyi Folium,* Morinda officinalis*,* Atractylodes macrocephala*,* Codonopsis* Radix, cornu cervi degelatinatum, Fructus Lycii, Dipsaci Radix, and* Eucommia ulmoides*. It was first introduced by Professor Yuankai Luo [7].

The effectiveness of the ZYP in treatment of threatened or recurrent miscarriages has been supported by clinical studies [7, 8]. It has also been shown to improve embryonic implantation rates in patients undergoing embryo transfer [9]. Collectively, these results strongly suggest that the ZYP can improve receptivity of the endometrium in both natural luteal cycles and ART cycles. These results suggest that the ZYP acts through HOXA10 to ameliorate COH-induced endometrial defects and that it improves uterine receptivity.

## 2. Materials and Methods

### 2.1. Animals and Reagents

Seventy-five sexually mature female and 40 male KM mice, aged 8-9 weeks, weighing 30–40 g, were housed at a density of five animals per cage at the Experimental Animal Center of Xinjiang Medical University, Urumqi, China. All animals were allowed free access to standard chow and water and kept in a 12 h light : 12 h dark schedule with constant temperature (21°C) and humidity (50%). The female mice were held in the facility over two estrous cycles prior to being placed on experiment. All experiments were approved by the Animal and Human Ethics Board of Xinjiang Medical University and conducted in accordance with Animal Research Committee Guidelines.

Pregnant mare serum gonadotropin (PMSG) and human chorionic gonadotropin (hCG) were purchased from Biosun (Shanghai, China), leuprolide (GnRHa) from Ipsen (Paris, France), and ZYP from Guangzhou Zhongyi Pharmaceutical Company (Guangzhou, China). TaqDNA polymerase, PCR primers, DAB chromogenic kits, PBS, poly-L-lysine, eosin dye, hematoxylin, rabbit polyclonal anti-mouse EMX2 antibody, rabbit polyclonal anti-mouse integrin *β*3 antibody, and rabbit polyclonal anti-mouse HOXA10 antibody had been ordered from Sangon Biotech (Shanghai, China). Chloroform, isopropanol, and ethanol were from EXCR (Shanghai, China). DNA markers (100 bp) were from SBS Genetech (Beijing, China). Tris, ammonium persulfate (APS), SDS, glycine, TEMED, and agarose were purchased from Sigma-Aldrich (St. Louis, MO, USA). PVDF was from Millipore (Billerica, MA, USA). Acrylamide, methylene-bis-acrylamide, and protein extraction reagent were from Active Motif (Carlsbad, CA, USA). Prestained protein standards were from BioRad (Hercules, CA, USA).

### 2.2. Generation of Animal Models

Seventy-five proestrus female mice were randomized into five groups of 15 each. The control group mice were only given saline, the model group 1 mice were given saline + PMSG + HCG, the model group 2 mice were given saline + GnRHa + PMSG + HCG, the treatment group 1 mice were given the ZYP + PMSG + HCG, and the treatment group 2 mice were given the ZYP + GnRHa + PMSG + HCG. COH of drug doses had been calculated according to Nagy et al. [10]. Dose of ZYP was calculated according to methods described by Fox et al. [11]. Saline 0.5 mL was fed by gastric perfusion; the ZYP was administered by gastric perfusion at a dose of 0.2 g/0.5 mL saline. Leuprolide (0.375 *μ*g), 10 IU PMSG, and 10 IU hCG were injected intraperitoneally per animal. Animals were handled as described by Nagy et al. [10]. The control and model groups 1 and 2 had been given 0.5 mL saline per day, while treatment groups 1 and 2 were given 0.2 g/0.5 mL ZYP solutions per day, for 13 consecutive days. Model group 2 and treatment group 2 were injected 0.375 *μ*g GnRHa per day for nine consecutive days. On the 9th day (except for the control group), groups were first injected with 10 IU PMSG, followed 48 h later with 10 IU HCG. Then two females from each group were placed with a male in a cage. The next day was considered to be the first day of pregnancy for females exhibiting a vaginal plug, and 75 females were killed on the second day of pregnancy. Their uteruses were collected, with one half used for measurement of mRNA level and the other half used for histology.

### 2.3. Hematoxylin-Eosin (H&E) Staining

The mouse uteruses were removed from 10% formalin, with different concentrations of ethanol for dehydration and increased transparency with xylene. Specimens were embedded in wax and then sliced into slices 4 *μ*m thick. The tissue chips were flated in a 40°C water bath, removed, mounted on the slide, and baked for 6 h. Slices were dewaxed with xylene and, with graded alcohol, stained with hematoxylin for 15 minutes and then washed with water. Slices were exposed to 1% hydrochloric acid in ethanol for 30 s, then washed in antiblue, stained with eosin alcohol for 2 min, and then washed. Slices were dehydrated with a different gradient alcohol, then rendered transparent with xylene, and mounted with neutral gum. Endometrial tissue morphological changes were observed under an optical microscope.

### 2.4. Relative RT-PCR

Total RNA was extracted from 100 mg of tissue using the Trizol reagent (Invitrogen, Carlsbad, CA, USA). The concentration and purity of RNA were determined using a spectrophotometer. The first strand of cDNA was synthesized as follows: 1 *μ*L total RNA, 1 *μ*L Oligo d(T)_15_, and 11 *μ*L DEPC water were mixed and incubated at 37°C for 5 min; after cooling on ice, 4 *μ*L 5x reaction buffer, 2 *μ*L dNTP, 1 *μ*L RNase inhibitor, and an appropriate amount of DEPC-treated water were added to adjust the volume to 19 *μ*L. After incubation at 37°C for 5 min, 1 *μ*L reverse transcriptase MmuLV was added and the mixture was incubated at 42°C for 60 min, followed by 10 min at 70°C to terminate the reaction, and the mixture was then cooled on ice.

GAPDH served as an internal reference for mRNA copy numbers of the target gene. Primer sequences were as the follows:

 Hoxa10 forward: 5′CCTTTCTCCCTCCCACACTC3′;
 Hoxa10 reverse: 5′ACAAACACCAAGCAAACAGACA3′;
 Integrin *β*3 forward: 5′GAATGATGTTGCCTTCCACTTT3′;
 Integrin *β*3 reverse: 5′TTCTTCCTTTCCCCAGTTATTG3′;
 EMX2 forward: 5′ACCTTCTACCCCTGGCTCAT3′;
 EMX2 reverse: 5′AATCCGCTTTGGCTTTCTG3′;
 GAPDH forward: 5′CCACCCATGGCAAATTCCATGGCA3′;
 GAPDH reverse: 5′TCTAGACGGCAGGTCAGGTCCACC3′.

The reaction was carried out in a 25 *μ*L system containing 2.5 *μ*L 10x PCR buffer, 1.5 *μ*L MgCl_2_, 0.5 *μ*L dNTP, 0.5 *μ*L upstream primers, 0.5 *μ*L downstream primers, 2.0 *μ*L cDNA, 0.5 *μ*L Taq DNA polymerase, and 17 *μ*L H_2_O. The PCR temperature parameters were as follows: an initial denaturation at 95°C for 5 min, and 30 cycles of 94°C for 30 s, 58.3°C for 30 s, and 72°C for 30 s, with a final template extension at 72°C for 10 min. Five microliters of PCR product was separated on 2% agarose gel by electrophoresis, stained with EB dye, and photographed using a gel imaging system.

### 2.5. Real-Time PCR

PCR amplification was conducted in a 25-*μ*L system that contains 2.5 *μ*L 10x PCR buffer, 1.5 *μ*L MgCl_2_, 0.5 *μ*L dNTP, 0.5 *μ*L upstream primers, 0.5 *μ*L downstream primers, 2.0 *μ*L cDNA, 0.5 *μ*L Taq DNA polymerase, and 17 *μ*L H_2_O. The PCR temperature parameters were as follows: an initial denaturation at 95°C for 5 min, and 40 cycles of 95°C for 15 s, 58.3°C for 30 s, and 72°C for 20 s. A melting curve was used to verify the specificity of PCR. CT value was used to calculate the target gene amplification using the relative quantitative 2^−ΔΔCt^ method. ΔCt = target gene Ct value − GAPDH Ct value, ΔΔCt = target gene ΔCt  − reference gene ΔCt, and the control group served as a reference. The amplification efficiency of each gene fell in the range of 90–110%. All genes were linearly amplified, and there was no nonspecific amplification.

### 2.6. Western Blotting

One hundred milligrams of frozen tissues was homogenized in 1 mL of ice-cold protein lysis buffer and the supernatant was used to examine the expression of HOXA10, integrin *β*3, and EMX2. Protein concentration was measured using the Bradford assay. Fifty micrograms of protein was separated on an SDS-PAGE gel by electrophoresis and transferred to a PVDF membrane. The membrane was blocked in 5% skim milk for 2 h and then incubated with the first antibody at 4°C overnight. Antibodies were diluted in 1x TBST buffer (1 : 250 for anti-integrin *β*3, 1 : 100 for anti-EMX2, 1 : 100 for anti-GAPDH, and 1 : 200 for anti-HOXA10). After a 3x wash in 1x TBST buffer, the membrane was then incubated with the second antibody (1 : 2000 diluted in 1x TBST buffer) at RT for 1 h. After 3 washes in 1x TBST buffer, the membrane was incubated with ECL reagent and exposed to a film. The intensity of protein bands was measured by using Gd-pro gel analysis software and normalized to that of GAPDH.

### 2.7. Immunohistochemistry

Fresh tissue was fixed in 4% paraformaldehyde, dehydrated, embedded in paraffin, and serially sectioned for immunohistochemical staining. Immunohistochemical staining was performed using SABC staining kits according to the manufacturer's instructions (Sangon, Shanghai). The sections were deparaffinized at 65°C, incubated twice in xylene, rehydrated in a graded series of ethanol, and soaked in 3% H_2_O_2_ in methanol at room temperature to inactivate endogenous peroxidase activity. After a step of microwave antigen retrieval, the sections were incubated three times in PBS for 5 min each and blocked in normal rabbit serum at room temperature for 15 min. Then the sections were incubated with primary antibodies (1 : 300 dilution) at 37°C for 1-2 h, followed by a further incubation at 4°C overnight. After 3 washes in PBS for 5 min, the sections were incubated with biotin-labeled secondary antibody in a working solution at 37°C for 20 min. After 3 washes with PBS for 5 min each, the sections were incubated with a horseradish peroxidase-labeled streptavidin working solution at 37°C for 20 min. After 3 washes with PBS for 5 min each, the sections were incubated with a DAB substrate solution, and the staining was closely monitored under a microscope. The sections were counterstained in a hematoxylin solution for 4 min, dehydrated, cleared, mounted, and examined under light-field microscopy. Positive staining was indicated by a brown precipitate. Staining was semiquantified and classified into four grades according to the color intensity. Five high-power fields (×400) were randomly selected from each slide and analyzed using a Biosens Digital Imaging System vl.6 automatic image analysis system (Biotop, Shanghai, China). The average optical density (OD) of intracellular positive staining was measured and the results were expressed as mean ± standard deviation.

### 2.8. Statistical Analysis

Data were analyzed using SPSS17.0 statistical software. The results are presented as mean ± standard deviation; comparisons among the five groups were performed using a *t*-test, with a bilateral *α* = 0.05 considered to be statistically significant.

## 3. Results

### 3.1. Generation of Mouse Models of COH

**Figure 1:**
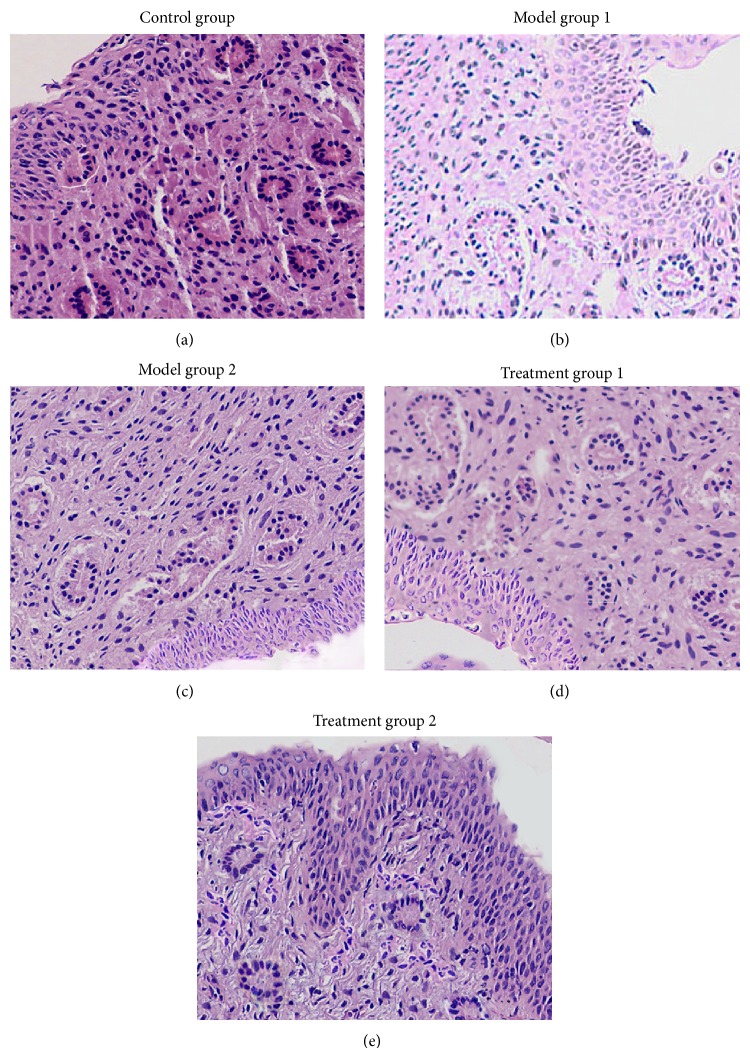
H&E staining of mouse endometrial sections. (a) Control; (b) model group 1, PMSG + HCG; (c) model group 2, GnRHa + PMSG + HCG; (d) treatment group 1, PMSG + HCG + ZYP; (e) treatment group 2, GnRHa + PMSG + HCG + ZYP.

Today's standard COH protocols involve cotreatment of GnRH agonist or antagonist with gonadotropins to prevent early increases in LH, and the use of GnRH agonists was found to be correlated with higher endometrial receptivity than the use of antagonists [6, 12]. Two gonadotropins, PMSG and hCG, were coinjected intraperitoneally (i.p.) to generate COH models. To suppress any premature LH surge, model group 2 also received an i.p. injection of the GnRH agonist leuprolide (GnRHa). H&E staining indicated that COH induced profound morphologic alterations in the endometrium (Figure 1). In the control group, many glands with relatively large lumens were visible, the stroma showed signs of mitotic activity. The stroma was compact, and the endometrium had thickened (Figure 1(a)). Model group 1 (which only received gonadotropin treatment) showed fewer glands and smaller lumens than the control group. The stromal and luminal epithelial cells were relatively small, irregular in shape, and densely compacted; the endometrium was thin (Figure 1(b)). Model group 2 showed fewer glands than the control group, but they did not have clear lumens. The stromal cells were small, irregular in shape, and densely compacted; luminal epithelia, glands, and stroma showed dyssynchrony characteristic of precocious maturation of the endometrium in COH (Figure 1(c)).

### 3.2. ZYP and the Glandular/Stromal Dyssynchrony of the Endometrium

Having established the mouse model of COH, the issue of whether ZYPs could ameliorate the glandular/stromal dyssynchrony of the endometrium was addressed. Mouse models were treated with the ZYP administered by gastric perfusion for 13 consecutive days. Treatment group 1 contained bigger gland cells and lumens and a thicker endometrium than model group 1 (Figure 1(d)). Treatment group 2 showed larger glands and gland lumens than model group 2, less compacted stroma, thicker endometria, and, most importantly, visibly synchronized glandular/stromal maturation (Figure 1(e)). In this way, ZYP can markedly ameliorate the precocious maturation of the endometrium induced by COH.

### 3.3. ZYP, COH, and Modulation of mRNA Levels for HOXA10, Integrin *β*3, and EMX2 in the Endometrium

**Figure 2:**
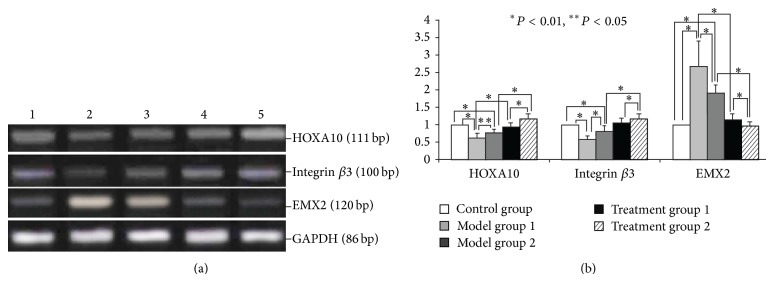
Mouse endometrial mRNA levels of HOXA10, integrin *β*3, and EMX2 in each group as analyzed by normal PCR and real-time PCR. (a) RT-PCR results. (1) Control; (2) model group 1; (3) model group 2; (4) treatment group 1; (5) treatment group 2. (b) Quantitative real-time RT-PCR results.

To investigate the mechanisms that may underlie the effects of ZYP on endometrial abnormalities induced by COH, the expression of HOXA10, a critical DNA-binding transcription factor involved in uterine organogenesis and embryonic implantation, was examined [13]. HOXA10, an important marker of endometrial receptivity, is expressed in both the epithelium and stroma of endometrium in a cyclical manner, with peak expression occurring during the window of implantation [14]. As shown in Figure 2(a), COH led to a decrease in HOXA10 mRNA levels as indicated by RT-PCR. Results were further confirmed by quantitative PCR (Figure 2(b)). HOXA10 controls the transcription of many genes, including integrin *β*3 and EMX2, which also serve as important markers for uterine receptivity [15–18]. Concomitant with the downregulation of HOXA10, integrin *β*3 mRNA levels also decreased (Figures 2(a) and 2(b)). In contrast to the positive effects on integrin *β*3, the transcription of EMX2 was reported to be suppressed by HOXA10 [16]. In accordance with the downregulation of HOXA10 by COH, results showed EMX2 mRNA levels to have increased (Figures 2(a) and 2(b)). ZYP treatment, then, notably promotes the expression of HOXA10. This can cause upregulation of the target gene integrin *β*3 and marked downregulation of EMX2 (Figures 2(a) and 2(b)).

### 3.4. ZYP Treatment Elevated Protein Levels of HOXA10 and Integrin *β*3 and Decreased EMX2 Protein Levels by Western Blotting in the Endometrium of COS Mice

**Figure 3:**
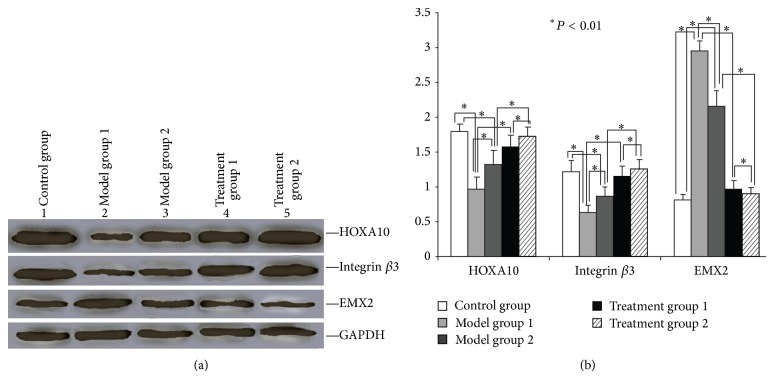
Mouse endometrial protein levels of HOXA10, integrin *β*3, and EMX2 in each group as analyzed by Western blotting. (a) Western blotting results. (1) Control; (2) model group 1; (3) model group 2; (4) treatment group 1; (5) treatment group 2. (b) Quantitative analysis of the Western blotting results.

To corroborate the regulation of HOXA10, integrin *β*3, and EMX2 expression by ZYP, the levels of the three proteins were examined using Western blotting. Consistent with the mRNA results, results showed that levels of HOXA10 protein were also decreased by COH (Figure 3). This decrease was accompanied by a downregulation of integrin *β*3 and an upregulation of EMX2 proteins (Figure 3). In accordance with the mRNA results, ZYP treatment led to increases in HOXA10 and integrin *β*3 protein levels and a decrease in EMX2 protein (Figure 3).

### 3.5. Changes in HOXA10, Integrin *β*3, and EMX2 Protein Levels Induced by ZYP Treatment as Indicated by Immunostaining

**Figure 4:**
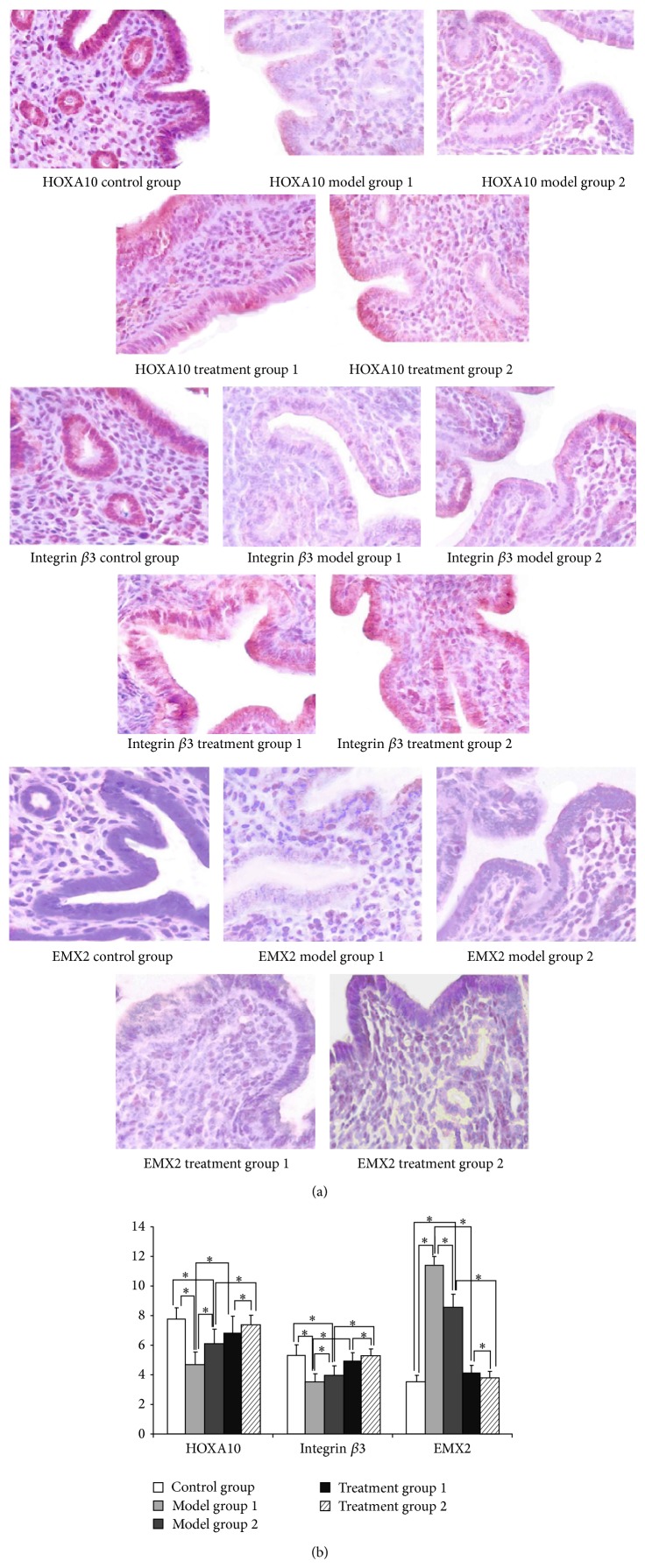
Endometrial immunostaining results for HOXA10, integrin *β*3, and EMX2 protein levels of staining in mouse endometrium in each group. (a) Immunostaining. (b) Quantitative analysis of endometrial immunostaining.

Immunostaining results confirmed that HOXA10 protein was expressed in the luminal epithelium, glandular epithelium, and cytoplasm of mesenchymal cells. Integrin *β*3 protein was located in endometrial luminal epithelial cells and the cytoplasm of epithelial cells. EMX2 showed very weak expression in uterine epithelial cells and epithelial cells (Figure 4). Results showed that expression of HOXA10 and integrin *β*3 protein had diminished but that of EMX2 proteins were augmented by COH (Figure 4) in the endometrium of the current mouse model. These alterations were ameliorated by ZYP treatment, expression of HOXA10, and levels of integrin *β*3 protein had increased. Levels of EMX2 proteins decreased after ZYP treatment (Figure 4).

## 4. Discussion

COH was developed to induce mature oocyte follicles, increasing the chances of pregnancy. It is the most commonly used protocol in ART [6]. Studies have shown that COH drugs stimulate production of large amounts of sex hormones and disrupt the hormones themselves. This leads to structural and functional changes in the endometrium ad uterine glands and development of the stroma of dyssynchrony. The endometrial implantation window and embryonic development are not synchronized, and this decreases endometrial receptivity and pregnancy rates [4, 5]. Here, 85% ovulation caused pinopode degradation 1-2 days in advance. Endometrial histological development occurs earlier than embryonic development [5]. van Vaerenbergh et al. compared endometrial estrogen and progesterone receptors in endometrial implantation window between natural luteal cycles and COH cycles. The index of COH cycles was found to decline, affecting the action of estrogen and progesterone on endometrial tissue development, synthesis, and secretion and the expression of genes related to endometrial receptivity related genes, all of which in turn affect embryo implantation [6]. Endometrial receptivity is essential to embryo implantation. A variety of cytokines, genes, and proteins bind to specific receptors on the endometrial cell surface. The endometrium can cause a series of changes in the positioning and implantation of the fertilized egg [3]. Changes in COH are closely associated with endometrial adhesion molecules, cytokines, timing of expression in the ultrastructure, interference of endometrial implantation window open, and blastocyst development of synchronization. In this way, it can affect endometrial receptivity [19].

Traditional Chinese medicine (TCM), considered gentle and safe, has long been used to treat infertility and miscarriages [20, 21]. It has also exhibited effectiveness in improving embryonic implantation rates in IVF [22]. In TCM theory, the kidney stores essence (or* jing*, which is the essence of* qi*) and dominates reproduction; so adequate kidney* jing* and strong kidney* qi* are vital to a successful pregnancy. The ZYP (“zishen” means “tonifying the kidney”; “yutai” means “nourishing fetus”) is a TCM formula that contains 15 herbs and other natural products developed by Professor Yuankai Luo to treat threatened or recurrent miscarriages by tonifying the kidney and increasing* yuan qi* [7]. Reproduction theory of TCM is used in ART. This work was performed to determine how to increase conception rates by tonifying the kidney and increasing* yuan qi* in molecular biology.

HOXA10 belongs to the Hox transcription factor family which functions as master regulators in directing embryonic development [22]. HOXA10 is involved in the embryogenesis of uterine epithelium, stroma, and muscle [23]. The expression of HOXA10 in the endometrium exhibits a cyclical pattern and is responsive for fluctuations in estrogen and progesterone levels, with maximal levels appearing during the window of implantation [14]. Targeted deletion of the* Hoxa10* gene was found to lead to uterine factor infertility in mice, primarily due to failure of embryonic implantation and aberrant decidualization [24]. Collectively, these studies suggest an essential role for HOXA10 in endometrial differentiation and embryonic implantation. HOXA10 is considered one of the most notable biochemical markers for endometrial receptivity [18]. The final cellular differentiation of the endometrium during the menstrual cycle resembles that observed during embryogenesis to a great extent, and* Hox* genes play central roles in determining cell fate during these processes. The downregulation of HOXA10 by COH was found to compromise cellular differentiation, causing precocious endometrial maturation.

HOXA10, a DNA-binding transcription factor, regulates the expression of many genes. Among these, integrin *β*3 and EXM2 also serve as important markers of endometrial receptivity [18]. Integrin *β*3 is an adhesion molecule involved in early interactions between the embryo and endometrium, and it plays an important role in embryonic implantation [25]. Endometrial expression of integrin *β*3 displays a cyclical pattern and appears abruptly on cycle day 20 on luminal and glandular epithelial cells, concomitant with the start of the implantation window [26]. HOXA10 directly controls the expression of the integrin *β*3 gene by binding to a 41 bp 5′-regulatory element [15], and the sex hormones also regulate the expression of endometrial integrin *β*3 through the HOXA10 pathway [26]. EMX2 is the human homolog of* Drosophila* EMS (empty spiracles) that contains a DNA-binding homeodomain involved in transcriptional regulation of animal development [27]. Emx2 has also been shown to regulate mammalian reproduction by limiting endometrial cell proliferation without affecting differentiation [28]. HOXA10 represses EMX2 gene expression via a direct interaction with a 150 bp regulatory region [16].

Because advanced endometrial maturation caused by COH contributes in large part to compromised endometrial receptivity and suboptimal pregnancy rates after IVF [3–5], one mechanism by which the ZYP improves endometrial receptivity is alleviation of COH-induced endometrial developmental defects. In support of this hypothesis, data collected using mouse models showed that ZYP in conjunction with GnRHa were able to ameliorate the precocious endometrial maturation and the resulting glandular and stromal dyssynchrony induced by COS to large extent. Treatment with ZYP alone was found to be less effective than ZYP/GnRHa cotreatment, most likely due to the ability of GnRHa to suppress premature surges in LH [6]. In this way, ameliorating the precocious maturation of the endometrium may represent the major mechanism whereby the ZYP improves uterine receptivity and pregnancy rates after IVF, and combined ZYP and GnRHa treatment may be used in ART to improve pregnancy outcome.

COH led to downregulation of HOXA10 expression and this downregulation was alleviated by the ZYP. It is therefore conceivable that the ZYP could act through the promotion of HOXA10 expression in order to maintain proper endometrial differentiation and ameliorate the aforementioned precocious maturation, thereby improving uterine receptivity and implantation rates. These results showed that COH induced downregulation of integrin *β*3 expression and upregulation of EMX2 expression and that the changes observed for both integrin *β*3 and EMX2 expression were alleviated by ZYP treatment. This was consistent with the HOXA10 results. Collectively, these results support a model wherein ZYP treatment upregulates the expression of HOXA10. Increased HOXA10 then drives the expression of many critical genes controlling endometrial receptivity, including integrin *β*3 and EMX2, which improves endometrial development and ameliorates the precocious maturation observed in COH mouse models.

In TCM, the kidney dominates reproduction, so pregnancy rates may be improved by treatments that enhance kidney* qi*. Many studies have confirmed the effectiveness of kidney-tonifying medicine in improving reproductive function. Li et al. reported in a mouse model that the kidney-tonifying medicine Bushen Zhuyu Decoction was able to increase numbers of ovarian follicles, endometrial glands, and stromal cells and promote endometrial hyperplasia (with the cells arranged in stratiform fashion) and elevate circulating estradiol and progesterone concentrations [29]. Two studies have shown that the use of kidney-tonifying TCM can reverse endometrial loss of leukemia inhibitory factor (LIF) and integrin *β*3 expression and improve uterine receptivity and pregnancy rates in mouse models of COH [30, 31]. A randomized controlled clinical study revealed that the use of kidney-tonifying medicine during COH cycles improved embryo quality and endometrial receptivity [32]. Hu et al. showed that kidney-tonifying medicine restored the expression of estrogen receptors in vaginal menopause and that it thus may serve as an alternative for hormonal therapy [33]. Kidney-tonifying medicine has also been found to reduce matrix metalloproteinase-2 (MMP-2) expression in endometriosis (presumably by improving the local endocrine environment), so as to reduce the invasion of ectopic endometrium [34]. The current results support the conclusion that kidney-tonifying medicines mitigate the precocious maturation of endometrium induced by COH, most likely via upregulation of the HOXA10 transcription factor. Collectively, multiple lines of evidence strongly suggest that TCM acts through multiple mechanisms to improve reproduction.

In conclusion, the present study showed that the ZYP was able to ameliorate advanced endometrial maturation via upregulation of HOXA10 in a mouse model of COH, thus providing initial molecular understanding of its underlying mechanism. These results also suggested that ZYP treatment could be a reliable approach to improving IVF outcomes.
